# Adaptation and psychometric study of the scale for the measurement of fear and anxiety of COVID-19 disease in pregnant women (AMICO_Pregnant)

**DOI:** 10.3389/fpubh.2023.1225822

**Published:** 2023-09-21

**Authors:** Francisco Javier Muñoz-Vela, Luciano Rodríguez-Díaz, Francisco Javier Fernández-Carrasco, Regina Allande-Cussó, Juana Maria Vázquez-Lara, Javier Fagundo-Rivera, Juan Gómez-Salgado

**Affiliations:** ^1^Department of Nursing, Faculty of Health Sciences, University of Malaga, Málaga, Spain; ^2^Regional University Hospital of Malaga, Málaga, Spain; ^3^Department of Nursing, Faculty of Health Sciences of Ceuta, University of Granada, Ceuta, Spain; ^4^Department of Nursing, University of Seville, Seville, Spain; ^5^Centro Universitario de Enfermería Cruz Roja, Sevilla, Ecuador; ^6^Department of Sociology, Social Work and Public Health, Faculty of Labour Sciences, University of Huelva, Huelva, Spain; ^7^Escuela de Posgrado, Universidad de Especialidades Espíritu Santo, Guayaquil, Ecuador

**Keywords:** psychometric properties, anxiety, fear, COVID-19, pregnancy, scale

## Abstract

**Objective:**

The aim of this research was to adapt and explore the psychometric properties of a specific scale to assess the levels of fear and anxiety of COVID-19 disease in pregnant women.

**Methods:**

An adaptation phase, by a panel of experts, and a psychometric descriptive cross-sectional study were carried out on the final version of the 16-item, self-administered AMICO_Pregnant scale. Univariate and bivariate analyses were carried out, followed by exploratory factor analysis (EFA) and confirmatory factor analysis (CFA). The consistency of the scale was assessed using the Omega coefficient and Cronbach’s Alpha.

**Results:**

With a sample of 1,013 pregnant women living in Spain and over 18 years of age, the mean age was 33 years. The scale showed a bifactor structure (anxiety and fear) that was confirmed with good fit parameters. Reliability was assessed in terms of internal consistency by calculating Cronbach’s Alpha coefficient (0.95) and McDonald’s Omega coefficient (0.94) as indicators of robustness of the scale’s reliability.

**Conclusion:**

The AMICO_Pregnant scale of 16 items with scores ranging from 1 to 10 is a valid and reliable tool to assess levels of anxiety and fear of COVID_19 in Spanish pregnant women. Pregnant women have shown moderate levels of anxiety and fear regarding the COVID_19 disease in the final phase of the pandemic.

## Introduction

In late December 2019, following the outbreak of the new coronavirus pneumonia in Wuhan, China, strict measures were taken to prevent the spread of the virus globally. These measures led to social distancing and closures at all levels internationally, though mainly in the service sector. The emergence of COVID-19 and its pandemic nature has exacerbated the feeling of fear and anxiety worldwide (1).

The initial confinement and individual protective measures to prevent transmission of COVID-19 disease masked some of the major risks associated with COVID-19 during pregnancy (2). However, based on currently available scientific information, an increased risk of maternal and perinatal morbidity and mortality associated with SARS-CoV-2 infection has been detected (3).

The clinical course of the COVID-19 disease varies from asymptomatic and mild to severe pathology with high morbidity and mortality (4, 5). These circumstances, together with the physiological changes that women undergo during pregnancy, have led to the inclusion of pregnant women as a group of special vulnerability to SARS-CoV-2 infection. Infections with other respiratory viruses, such as influenza virus or other types of coronavirus, have also been associated with complications during pregnancy (2).

Most people infected with COVID-19 experience mild to moderate symptoms (fever, fatigue, sore throat usually with odynophagia, cough, and dyspnoea). However, for pregnant women, the most recent studies describe an increased risk of hospitalisation and ICU admissions (6–8) despite the fact that the occurrence of vertical transmission is extremely rare (9, 10). Increased risk of pregnancy-related pathologies such as pre-eclampsia, threatened preterm birth, or low birth weight have also been reported (11, 12), as well as respiratory infections or pneumonias that are associated with adverse outcomes and increased maternal and infant morbidity (13).

It is in this context that there has been a historic increase in negative emotions such as fear or anxiety as a psychological response to cope with the illness (14) or to deal with depressive and anxious symptomatology (15, 16).

Some research has pointed to the relationship between prenatal maternal stress and anxiety levels and compromised optimal development of the hypothalamic–pituitary–adrenal HPA axis, limbic system, and prefrontal cortex (17, 18). Fear and concern about the disease affect the behaviour of the most susceptible individuals, as no one wants to be infected with a virus that poses a high risk of death (19).

According to the Centres for Disease Control and Prevention, fear and stress related to COVID-19 have led to emotional disturbances such as sleep and eating disorders, worsening of symptoms in psychiatric pathologies, and increased substance use that may precipitate mental disorders (20).

Fear is generally recognised as an adaptive protective mechanism in humans and animals that is fundamental for survival. Fear does not exist permanently, as it decreases in the absence of an adverse stimulus (21). However, if the stimulus is sustained over time or becomes characteristic of the individual, it may predispose to physical illness and/or psychological disorders, or even aggravate previous mental pathologies (22).

Due to the unprecedented global reach of the pandemic in the short term, with many different protective measures (23, 24), the fear of coming into contact with people at risk of being infected with COVID-19 has disrupted normal coexistence and led to people being at increased risk of mental health problems, such as fear of infection, uncertainty, stress, anxiety disorders, and sleep problems among others (19, 25). Fear of the COVID-19 disease is therefore considered to be one of the main triggers of mental health problems nowadays (26).

This new social reality has led, according to several reports, to increased levels of anxiety, fear, and depression among the general population, health professionals, and vulnerable groups (26–28). In general terms, the literature suggests that the impact of the pandemic and its restrictions has had a broad, substantial, and potentially long-lasting impact (29). In this sense, adolescents and young adults have been reported to experience increased stress, anxiety, and depressive symptoms due to the pandemic. The effects on these groups can be attributed to factors such as school disruption, social isolation, uncertainty about the future, and potential economic hardships upon entering the workforce (30, 31). However, studies on the influence of the COVID-19 pandemic on the mental health of pregnant women are scarce despite the fact that such research has shown a substantial increase in symptoms of perinatal depression and anxiety especially in postpartum women through the use of non-specific instruments, which suggests the need for further research and interventions in the perinatal period (32, 33). The limitations imposed by protective measures intensified the stressors that influence women’s mental health during pregnancy (34). Given that this is a period of particular vulnerability to experience mental disorders (35), approximately 10–20% of women suffer from a mental health problem during the prenatal period (36). Research carried out in different countries such as the United States, Canada, Italy, Turkey, Greece, and China have estimated an increase in the prevalence of anxiety and depression among pregnant women (37, 38). These results are in line with research showing the pandemic period as a precursor of psychopathological symptomatology (15).

These studies, however, do not address the relevance of identifying the negative feelings, emotions, or thoughts that pregnant women have had during the pandemic. This would be essential not only for prevention, treatment, or diagnosis, but also for designing an individualised plan for the management and treatment of these mental conditions (39).

Among the most widely used instruments for measuring negative emotions such as fear is the FCV-19 (fear of COVID-19) scale by Ahorsu et al. (40). It was designed with a unidimensional structure composed of seven items, which presented a total correlation of the corrected items between 0.47 and 0.56 and which were confirmed with significant and high factor loadings (0.66–0.74) and reliability values such as internal consistency (alpha = 0.82) (40, 41), having been validated with an older adult Iranian population and showing adequate psychometric properties. Later, FCV-19 was validated in several countries: the United States (42), Paraguay (43), Turkey (44), Saudi Arabia (45), or Spain (46), while maintaining its unidimensional factor structure, seven-item composition, and adequate psychometric properties. Among the research that has used the FCV-19 scale for the assessment of fear in the population is the study by Li et al. (47) on an older adult population (*n* = 139: mean age: 71.73), that by Mistry et al. (48) (*n* = 1,032; age > 60), and the one by Moussa et al. (49) in Saudi Arabia (*n* = 969; mean age: 35.5) on a group of nurses. This tool is also commonly used to assess anxiety levels in pregnant women, although no study has reported its psychometric validation for this specific population (50).

On the other hand, the study by Gomez-Salgado et al. (51) developed the Anxiety and Fear of COVID-19 Assessment Scale (AMICO, for its acronym in Spanish), based on the original 10-item version of the FCV-19 scale and incorporating eight new items assessing the specific presence of COVID-19 anxiety. The tool was developed and validated during the third wave of the COVID-19 pandemic in Spain, already in a situation of de-escalation from the initial confinement. For this purpose, a population of 1,036 participants over 18 years of age living in Spain was assessed. Following its original publication, a second research study confirmed that a two-dimensional structure of 16 items was obtained, as well as two factors that explained 64.8% of the variance (52). The reliability study gave a total Cronbach’s alpha value of 0.92 for factor 1 (Anxiety) and 0.90 for factor 2 (Fear). The AMICO scale has been validated with different population groups: theolder adult (53), nurses (54), adult general population (55), or the adult general population in United Kingdom (56). Eventually, the research team behind the design of the AMICO Scale established the following cut-off points: low level of fear and anxiety with a score from 0 to 4.31 points; intermediate level, from 4.32 to 6.4 points; and high level, with a score above 6.4 points (51).

In this context, the need arises to design a tool that specifically measures the fear and anxiety caused by the pandemic in a group with different psychological characteristics from the population groups already studied. One of the reasons why the current literature about COVID-19 pays little attention to fear and anxiety of COVID-19 is the lack of a specific psychometric instrument. Therefore, the aim of this research was to adapt the scale, based on the consensus of a panel of experts, and to study the psychometric properties of the AMICO_Pregnant scale as an instrument to measure the levels of fear and anxiety of COVID-19 in the Spanish pregnant population.

## Methods

### Design

Cross-sectional study of psychometric analysis, in two phases: (1) adaptation of the AMICO scale to a sample of Spanish pregnant women by a panel of experts; and (2) descriptive cross-sectional psychometric validation study.

### Participants

To achieve the aim of the research, according to Epstein et al. (57), two different groups of participants were formed: on the one hand, the panel of experts who agreed to participate in the study consisted of 10 professionals and researchers from different Spanish universities, with an academic level of Doctor or Official Master’s Degree and whose areas of knowledge were obstetrics, public health, or psychology; one of the experts was also pregnant at 28 weeks of gestation. On the other hand, for the descriptive cross-sectional study, data were collected about the population of pregnant women in Spain; the number of births recorded in Spain during the year 2021 amounted to 337,380 births according to the Spanish National Statistics Institute (INE, for its acronym in Spanish). The required sample size was calculated considering a confidence level of 95%, for a maximum sampling error of 5%, and it was 385 participants (58). However, the final sample obtained was 1,013 pregnant women.

### Variables

The study variables included were: (1) socio-demographic (age, place of residence, marital status and cohabitation situation, level of education, occupation, and employment sector); (2) COVID-19 related (two items: vaccination status in relation to COVID-19 and contact with the disease); obstetric (six items: weeks of gestation, parity, type of gestation, type of conception, type of prenatal control, and changes in the birth plan); (3) and scale variables (16 items: AMICO_Pregnant scale). The AMICO_Pregnant scale has a two-dimensional structure of 16 items. The reliability study gave a total Cronbach’s alpha value of 0.95 and Omega’s value of 0.94. The range of scores is from 1 to 10, where 1 is the lowest level and 10 the highest possible level.

Eventually, the final data collection tool contained a total of 33 items, considering all the questions related to socio-demographic variables, those related to the COVID-19, and the scale.

### Procedure

#### Phase 1: adaptation of the AMICO scale

The Spanish version of the AMICO scale was adapted to the population of pregnant women by a panel of 10 experts, using the Google Forms© application (Google, Mountain View, CA, United States) and the two-step Delphi technique:

*First step:* A first round to ascertain the experts’ opinion on the need to include new items in the questionnaire, as it was intended to assess anxiety and fear in the pregnant population. Therefore, they were freely encouraged to write down the strictly necessary items to be inserted.
*Second step*: A second round with the new items identified in the previous step; the opinions and final consensus obtained were analysed.

To determine the final items of the adapted scale, the content validity ratio (CVR) was calculated (59, 60), based on the Lawshe’s Content Validity Ratio. Items with a CVR score of at least 0.8 were considered adequate.

The questionnaire version finally agreed by the group of experts was piloted on a set of 300 pregnant women selected during the pregnancy control consultation at a regional University Hospital. The mean gestational age was 37 weeks (SD = 3.44), and the mean age of the women was 32 (SD = 5.64). A total of 298 surveys were obtained with no incidents, and no pregnant woman reported the need to modify the wording of any of the items.

#### Phase 2: descriptive cross-sectional psychometric validation study

Between March and July 2022, data collection was carried out using the designed tool, which contained all the variables and final items agreed in the previous phase by the panel of experts.

The questionnaire was disseminated by mass mailing of a link and QR code linked to a GoogleForms form to the email addresses of women with an ongoing pregnancy obtained from an updated self-registration database of a free national magazine with content and newsletters related to pregnancy and childcare (*Mi Bebé y Yo*). In addition, other organisations related to perinatal care, midwifery teaching units in three Spanish provinces, health centres in these same three provinces, and public and private hospitals also participated in the dissemination of the survey.

The corresponding QR code or direct link redirected to a survey created using the GoogleForms© application. Once the pregnant woman accessed the questionnaire, information was displayed on the legal, consent, and confidentiality conditions for accessing the questionnaire, as well as an email address as a means of direct communication for consulting and exercising rights and duties in terms of data confidentiality, and to make queries about the study.

### Data analysis

Using the SPSS v.28 (IBM, Armonk, NY, United States) statistical software univariate and bivariate analyses of the data were performed. For the latter, the normality test of the distribution was previously carried out using the Kolmgorov-Smirnow test, which determined the non-normality of the distribution, so non-parametric tests were used to study the contrast hypothesis: Spearman’s Rho, Mann–Whitney U, Kruskal-Wallis, and Kendal’s Tau-b for the study of correlations between variables or between variables and the total score of the AMICO scale. An exploratory factor analysis (EFA) was used to study the dimensional structure of the scale and to determine the percentage of variance explained. Previously, the Kaiser-Meyer-Olkin (KMO) statistic and Bartlett’s test of sphericity were calculated. Initial factors were extracted from the correlation matrix using the Principal Component Analysis method and Varimax rotation.

Then, construct validity was assessed by means of a confirmatory factor analysis (CFA). To assess the goodness of fit of the confirmatory models using the AMOS software (61), incremental Fit Indices were run to assess the improvement of the proposed model in relation to a base *Comparative fit index* (CFI) model; *Tucker-Lewis index* (TLI); Normalised Fit Index (NFI); (sample size > 100, value > 0.93). The Root Mean Squared Error of Approximation (RMSEA, where values ≤0.08 indicate a good fit) and Standardised Root Mean Squared Residual (SRMR), where values ≤0.08 indicate a good fit indices were also calculated.

Regarding the reliability study, the internal consistency between the items was assessed by calculating Cronbach’s Alpha coefficient and also McDonald’s Omega coefficient as an indicator of robustness of the scale’s reliability.

### Ethical aspects

The Declaration of Helsinki of 2013 was taken into consideration for this study (62) and explicit permission was obtained from the participants through an informed consent for the confidential use and processing of the data in accordance with the Law on Data Protection and Digital Rights (Law 3/2018 5 December). Data have been kept by the research team. Likewise, the study obtained the approval of the Biomedical Research Ethics Committee.

All participants were requested an informed consent by telematic means, in online format, or in person, verbally and in writing. In it, they were informed of the purpose of the study, as well as of the possibility of participating in the study on a completely voluntary basis, while ensuring the confidentiality of data at all times. They were also informed that participation or non-participation would have no positive or negative repercussions and that they could drop out of the study at any time without any type of effect. After reading this information and in order to give informed consent, they had to select the option of being 18 years of age or older and ‘YES, I GIVE CONSENT’ to participate in the current research entitled ‘Assessment of the Impact of the COVID-19 Pandemic on the Emotional Well-Being and Psychological Adjustment of Pregnant Women’, which allowed them to access the instrument.

## Results

### Adaptation of the scale

The 16 items of the original AMICO scale were assessed by the panel of experts in a first round. Improvements were made to the wording of the items to address the pregnant population, but no items were modified as to their concept or description. In addition, the experts proposed the wording of eight new items. In a second round, the preliminary version of the questionnaire, finally composed of 24 items, was subjected to a new assessment by the research team with the aim of evaluating its applicability and agreeing on the final version to be tested. Thus, the experts used a five-point Likert scale to assess the adequacy of each item: not at all relevant (one point), somewhat relevant (two points), quite relevant (three points), very relevant (four points), and most relevant (five points).

The final scale was made up of those items with a CVR score of at least 0.8, making a total of 16 (Supplementary material 1).

### Descriptive analysis

During a period of 4 months, a total of 1,013 surveys were completed by pregnant women with a mean age of 33.38 years, with a standard deviation of 5.2 years. A total of 47.5% were married, 41.1% were in a stable relationship, and 11.5% reported having no partner (Table 1).

**Table 1:** Summary of the results of descriptive analysis and contrast hypothesis.

	Total sample (*n* = 1,013)	AMICO mean (based on 10)	Contrast Hypothesis^*^
Age	33.483 (5.298)		*p* = 0.919^a^
Mean (SD)			
Marital status			*p* = 0.326^b^
Married	481 (47.5%)	5.003	
In couple	416 (41.1%)	4.706	
Single	105 (10.4%)	5.211	
Divorced	7 (0.7%)	3.885	
Widower	4 (0.4%)	4.786	
Weeks of gestation		5.04	***p* = 0.000**^**a**^
Median	37		
Mean	32.78 (8.328)		

^*^Non-parametric statistical contrast: ^a^Tau Be Kendall, ^b^Kruskal-Wallis.

Concerning the current pregnancy, 3.4% of the pregnant women were in their first trimester of pregnancy, 13.6% in their second one, and the majority were full term 83% (median = 37 weeks). Over 58.8% were primigravidae and 98.7% had a singleton pregnancy. Pregnancy control was mainly low risk 52.1%.

The bivariate analysis showed no statistically significant relationship between the AMICO_Pregnant scale and the age or marital status variables. However, the correlational analysis did show statistically significant differences regarding the AMICO_Pregnant scale scores and weeks of gestation (Table 1).

The mean total score of the AMICO_Pregnant scale was 5.04 points (SD = 2.36). The minimum score was one point, and the maximum score was 10 points. Skewness scores ranged from −0.80 to 0.92, and kurtosis scores ranged from −1.07 to −0.36, for all scale items and the total mean score (Table 2).

**Table 2:** Descriptive statistics of each item of the scale and for the total score.

	Minimum score	Maximum score	Mean	Standard deviation	Skewness score	Kurtosis score
AMICO_1	1	10	4.72	2.632	0.222	−0.937
AMICO_2	1	10	4.76	2.719	0.211	−1.052
AMICO_3	1	10	6.36	2.946	−0.402	−1.071
AMICO_4	1	10	6.39	2.905	−0.364	−1.079
AMICO_5	1	10	4.58	2.751	0.310	−1.001
AMICO_6	1	10	4.64	2.784	0.297	−1.027
AMICO_7	1	10	3.26	2.504	0.925	−0.124
AMICO_8	1	10	4.83	3.000	0.261	−1.192
AMICO_9	1	10	4.39	2.829	0.428	−0.969
AMICO_10	1	10	4.06	2.686	0.535	−0.786
AMICO_11	1	10	6.25	2.725	−0.337	−0.908
AMICO_12	1	10	7.33	2.849	−0.805	−0.599
AMICO_13	1	10	5.37	2.882	0.027	−1.172
AMICO_14	1	10	5.99	2.980	−0.199	−1.200
AMICO_15	1	10	4.66	2.947	0.324	−1.092
AMICO_16	1	10	3.45	2.644	0.860	−0.361
Total mean score	1	10	5.04	2.36	0.076	−1.021

### Psychometric analysis

#### Content validity

To determine the degree of face validity of the scale, each of the questions of the AMICO scale (for the general population) was subjected to the judgement of 10 experts in the field of obstetrics, public health, and pregnant women who assessed the adequacy of the items for pregnant women. The experts evaluated the wording of the items included in the instrument by means of the Delphi method, using a five-point rating: (1) not at all relevant, (2) somewhat relevant, (3) quite relevant, (4) very relevant, and (5) most relevant.

In the first round of the Delphi panel, of the 16 initial items of the original scale, 11 items were proposed to be modified in their wording. In the second round, these 11 items were again subjected to the Likert scale described above in order to assess the adequacy of their inclusion in the new scale, by calculating the RVC (Table 3).

**Table 3:** Content validity by panel of experts.

	Experts’ scores (from 0 to 5 points)										Total	RVC
Number of expert	1	2	3	4	5	6	7	8	9	10		
Item 2—Thinking about COVID-19 causes me DISTRESS.	5	5	5	5	5	5	5	5	5	5	50	1
Item 3—I am very worried about getting COVID-19 FOR MY HEALTH AND MY BABY’S HEALTH.	5	5	5	5	5	5	4	5	5	5	49	0.80
Item 4—The COVID-19 disease can be life threatening, and this worries me FOR MY HEALTH OR MY BABY’S HEALTH.	5	5	5	5	5	5	5	5	5	5	50	1
Item 5—I GET VERY NERVOUS when I think about COVID-19.	5	5	5	5	5	5	5	5	5	5	50	1
Item 7—I have trouble sleeping if I think I might get COVID-19.	5	5	5	5	5	5	5	5	5	5	50	1
Item 8—My pulse races if I have been in close contact with someone AT RISK OF being infected.	4	5	4	5	5	5	5	5	4	5	49	0.80
Item 9—Contradictory information about COVID-19 in the media and social networks makes me feel anxious.	5	5	5	5	5	5	5	5	5	5	50	1
Item 10—I am assaulted by negative thoughts when I hear or read news related to COVID-19.	5	5	5	5	5	5	5	5	5	5	50	1
Item 11—I am worried that a family member or friend might contract COVID-19.	5	5	5	5	4	5	5	5	5	5	49	0.80
Item 15—I FEEL sad or weak when I think about the disease and the possibility of infecting myself or my baby.	5	5	5	5	5	5	5	5	5	4	49	0.80
Item 16—I FEEL anxious about leaving home, or thinking about it, to fulfil my daily duties (work, family, etc.) due to the current pandemic situation.	5	5	4	5	5	5	5	5	5	5	49	0.80

#### Construct validity

An exploratory factor analysis (EFA) was conducted in order to identify scale-specific internal factors. The Kaiser-Meyer-Olkin (KMO) sample adequacy statistic was estimated and found to be 0.962, and Bartlett’s test of sphericity was found to be statistically significant: *X2* = 19100.292; *p* < 0.000. In both cases, the evidence confirmed the adequacy of the factor analysis.

The EFA showed the two-dimensional structure of the questionnaire and the extraction of two factors that could explain 78.935% of the total variance. The first dimension consisted of eight items with factor loadings ranging from 0.873 to 0.93. These items describe situations related to the presence of anxiety in relation to one’s own pregnancy or how COVID-19 would affect the newborn. The second dimension consisted of eight items with a minimum factor loading of 0.76 and a maximum factor loading of 0.87. These items refer to situations associated with the level of fear, concerning one’s own pregnancy or how COVID-19 would impact the newborn. No items were removed as the factor loadings were always well above 0.60 (Table 4).

**Table 4:** Exploratory factor analysis.

	Anxiety	Fear
ITEM_1		0.87
ITEM_2		0.86
ITEM_3		0.83
ITEM_4		0.80
ITEM_11		0.78
ITEM_12		0.76
ITEM_13		0.83
ITEM_14		0.84
ITEM_5	0.93	
ITEM_6	0.93	
ITEM_7	0.83	
ITEM_8	0.85	
ITEM_9	0.87	
ITEM_10	0.89	
ITEM_15	0.84	
ITEM_16	0.79	

Subsequently, a confirmatory factor analysis (CFA) was carried out on the AMICO_Pregnant scale data to determine the validity of the factor structure that defines each of the dimensions. Seven indices were used to assess the fit of the model to the data.

**Figure 1:**
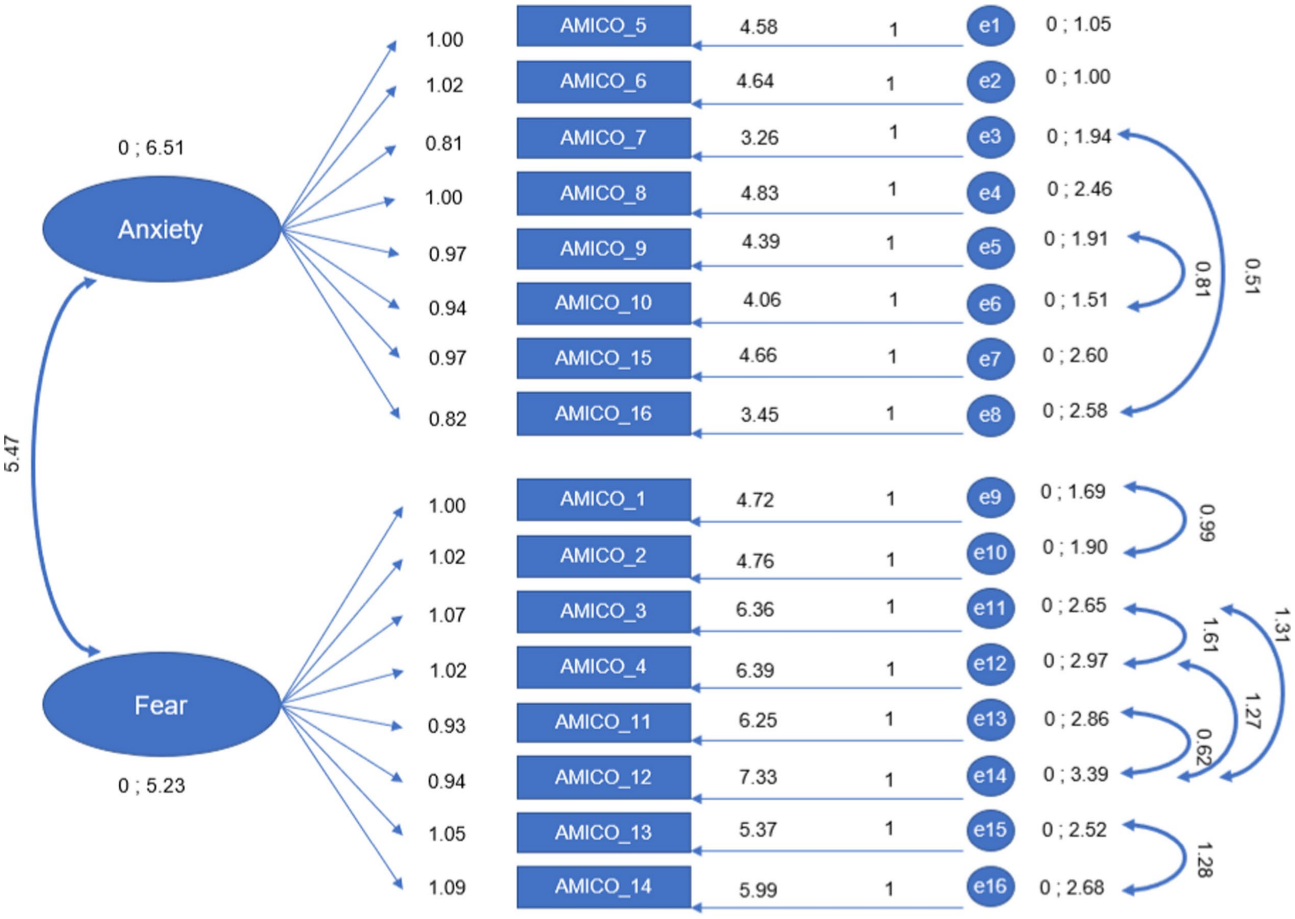
Confirmatory factor analysis.

The modification indices indicated the existence of feedback loops or correlated errors between items (between 7 and 10; 9 and 10; 1 and 2; 3 and 4; 3 and 12; 4 and 12; 11 and 12; and 13 and 14), which significantly improved the fits to acceptable levels (CMIN/DF = 12.030; NFI = 0.942; TLI = 0.932; CFI = 0.946; RFI = 0.926; SRMR = 0.037; and RMSEA = 0.104; Figure 1).

#### Reliability

The reliability indices measured with both Cronbach’s Alpha and McDonald’s Omega were 0.95 and 0.94, respectively.

#### Levels of anxiety and fear and their distribution across the sample

**Figure 2:**
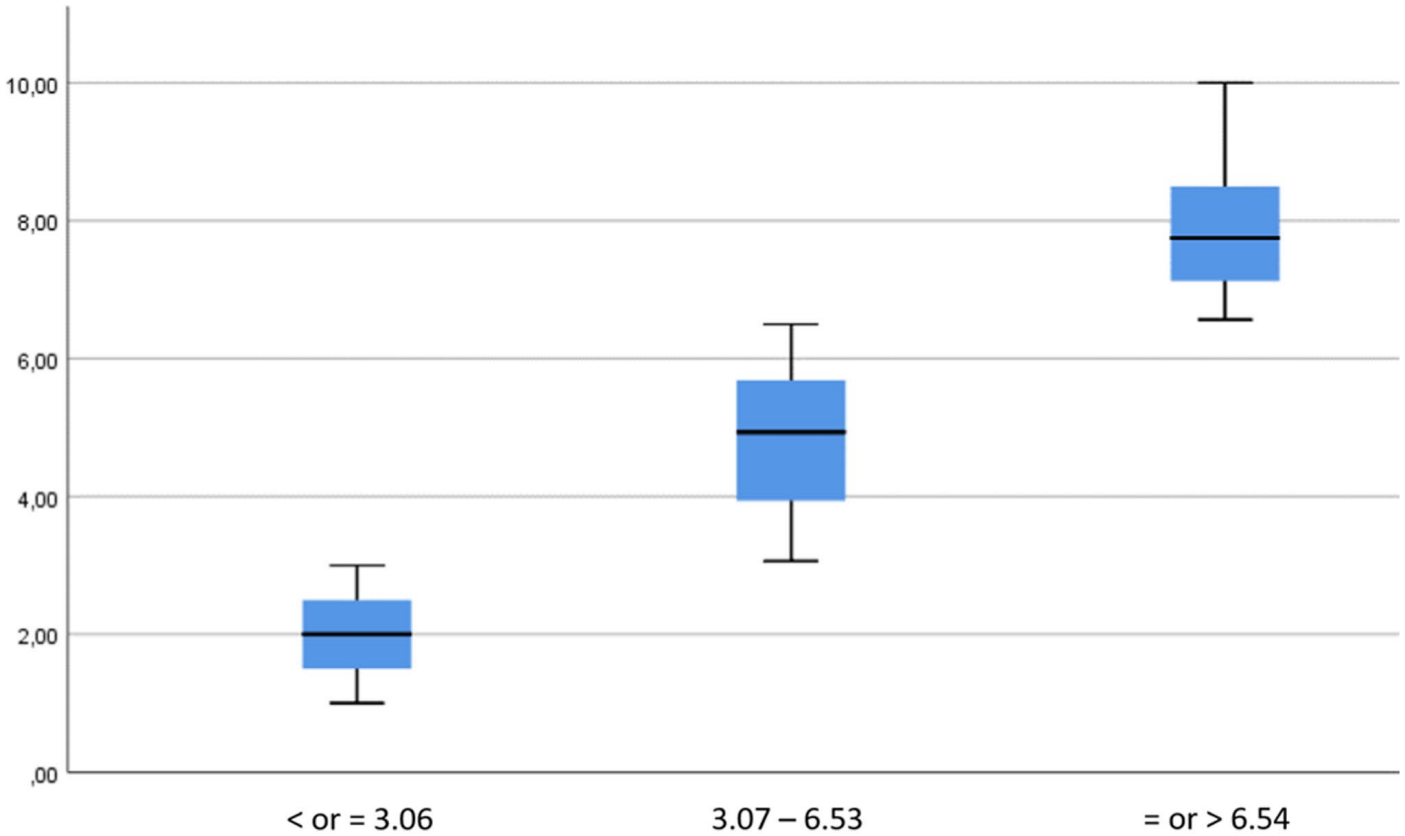
Proposed levels of anxiety and fear in the pregnant population.

The mean score of the AMICO_Pregnant scale was 5.04 (SD = 0.075), with a range of scores from 1 to 10. The study of percentiles and quartiles in relation to the distribution of the mean scores of the AMICO_Pregnant scale helped to identify three differentiated levels of fear and anxiety. In this sense, the following relationship of levels was proposed for the AMICO_Pregnant scale: low level with scores from 0 to 3.06 points; intermediate level, from 3.07 to 6.53 points; high level, a score of more than 6.54 points (Figure 2).

The statistical significance of the significant differences between each pair of levels analysed always gave a value of *p* = 0.001, using the Mann–Whitney U statistics; therefore, significant differences were found between the levels identified, and thus, they were independent of each other.

## Discussion

The present study aims to provide more information on the psychometric properties of the AMICO_Pregnant Scale based on a scale designed for the general Spanish population (52).

Motivated by the special characteristics of the pregnant population, it was considered that, despite validations in different population groups, it was likely that the specific characteristics of pregnant women were not being represented, so it might be advisable to develop a specific adaptation of the questionnaire for this population group, whose behaviour is very different from the general population due to the disease itself together with the concern associated to carrying a baby.

Given that levels of fear and anxiety are determined both by the characteristics of the pandemic context and by the presence of an intrauterine foetus (16), a very large and diversified sample of pregnant women across the country was chosen. This was done with the aim of covering different periods of the pandemic and to obtain more information on the effect size.

The exploratory factor analysis showed the maintenance of a two-factor structure with two interrelated factors: fear and anxiety underlying the data and 16 items as a result of their high factor loadings. These results offered a structure consistent with previous two-factor studies obtained from a general population by Gomez-Salgado et al. (52), which explained a total variance of 64.8%, or in the case of research involving a sample of older adult people, which showed a total variance of 66.6% (53). The results of the study showed a distribution that explained a total variance of 78.935%. Therefore, from the results obtained it can be extracted that the items of the scale presented a good level of internal consistency, which is indicative of the reliability of the instrument in a sample of pregnant women.

Regarding the reliability of the instrument, the Cronbach’s Alpha index (0.95), which measures the correlation of the items within the questionnaire, has been considered for years the most suitable indicator as it gives a single value of consistency. A value between 0.8 and 0.9 is considered a very good level by researchers, and a value >0.9 is considered optimal (63). However, some researchers have indicated a maximum value of 0.90; higher values could indicate redundancy or duplication, meaning that several items are measuring exactly the same element of a domain or construct (64). In this regard, other studies point to the need to consider the impact of correlated errors modelled from modification indices and their impact on reliability indices together with the absence of tau-equivalence (65), which may lead to overestimation of reliability estimates (66).

In view of this, McDonald’s Omega coefficient is considered a more suitable alternative, as its use is not as restrictive and is more convenient to be applied in structural equation models (66). In this sense, this study provides the McDonald’s Omega coefficient (0.94) as an added reliability data, which is optimal and provides greater robustness to the reliability study of the AMICO_Pregnant instrument. Along these lines, both the study by Velez-Moron et al. (53) in the validation of the AMICO scale in the older adult population and the one by Morgado-Toscano et al. (56) in validating AMICO in the United Kingdom general population showed similar or slightly lower values for both Cronbach’s Alpha coefficients (0.94 and 0.96, respectively) and McDonald’s Omega coefficients (0.91 and 0.92, respectively), and very similar to the results reported by Gomez-Salgado et al. (48) in the general Spanish population, which showed a Cronbach’s Alpha coefficient value of 0.92. The McDonald’s Omega coefficient was not calculated in the research by Gomez-Salgado et al. (51), despite the modifications made to the wording during the design phase of the scale.

However, other scales measuring fear in samples of non-pregnant women showed clearly lower internal consistency values, such as the FCV-19 scale developed by Ahorsu et al. (40), which showed lower values than those found in AMICO_Pregnant both in its development (α = 0.82) and in its different validations, the one by Li et al. (47) on a Taiwanese sample using the FCV-19 scale (α = 0.79), by Mistry et al. (67) in the Bangladeshi population (α = 0.91), or by Pakpour et al. (63) in the Iranian population (α = 0.91).

In this vein, the mean score obtained for anxiety and fear levels of COVID-19 in pregnant women showed to be slightly lower (mean = 5.04, SD = 2.36) for a mean age of 33 years compared to other AMICO scale results in previous studies on the general population. Allande-Cussó et al. (55) obtained a mean score on the AMICO scale for the general Spanish population of 5.41, SD = 1.83, with a range of scores between 1.22–10 during the months of October and November 2020, a time when restrictions were particularly severe to prevent the spread of the virus. This research carried out in Spain obtained a sample of 56.3% women with a mean age of 48.11 (SD = 15.11), with higher levels of anxiety and fear among women than among men (mean AMICO score for women 5.3, and for men 5.0). Reasons for the lower levels of fear and anxiety in the present study than in previous research in women with or without pregnancy may include the timing of data collection, i.e., 2 years apart, substantially different levels of vaccination, a lower perception of the dangerous nature of the disease based on decreased hospital admissions, ICU admissions, and deaths resulting in an estimated vaccination effectiveness versus hospitalisation in the 18–39 year age group of 49%, and no data on deaths (68).

In relation to marital status, previous studies have shown contradictory results at different stages of the pandemic. These findings may be due to the influence of social isolation. Previous research with the AMICO scale showed a significant relationship with levels of anxiety and fear, with married people showing higher levels (5.55), followed by cohabiting, widowed, and divorced people (5.10), and with single people showing lower levels of anxiety and fear (4.15) in the study by Allande-Cussó et al. (55) on the Spanish population. However, the present study was unable to identify significant differences in the levels of anxiety and fear based on marital status. In this case, this could be justified by the characteristics of the sample, with 89.1% of the sample being in a couple situation compared to 10.9% who declared to be without a partner.

The present study has some limitations. On the one hand, the non-probabilistic sampling through which the sample was obtained could have affected the generalisability of the data. On the other hand, data collection was mainly telematic, which may have generated an accessibility bias as certain social groups of particular vulnerability may have been excluded from access to the questionnaire. Furthermore, it would be advisable to carry out further studies to validate the psychometric properties of the new scale and to implement criterion validity analyses by studying the convergent validity and calculating the ROC curve, as well as the values of sensitivity, specificity, positive predictive validity, and negative predictive validity. Also, there is a need to continue researching the long-term consequences of COVID-19 to assess the possible damage on mental health (69).

Concerns and expectations in relation to pregnancy and childbirth have changed due to COVID-19 disease (70, 71). Although the literature suggests that the infection is not currently particularly dangerous for pregnant women (72), the research undertaken seeks to understand the scope of fear and anxiety about COVID-19 infection in a group that is particularly vulnerable or predisposed to mental health disturbances. These findings are not only valuable for predicting fear and anxiety in pregnant women, but they also have the potential to guide their application in clinical settings and future research. By identifying key variables that contribute to these emotional states, this tool could help health professionals to assess and address psychological well-being during pregnancy. Additionally, the information obtained in this study underlines the importance of developing comprehensive birth and delivery plans that consider real-life situations, such as possible future pandemics. These plans should be tailored to the individual’s circumstances and consider factors such as public health measures and access to healthcare services to ensure a positive and satisfactory experience for both the woman and her support network.

The conclusion of the study is that AMICO_Pregnant scale has been adapted as a tool for the assessment of anxiety and fear of the COVID-19 disease, showing adequate construct validity and reliability in the measurement of anxiety and fear in Spanish pregnant women. It consists of 16 items with a 10-point Likert-type response. Therefore, the scale could be used in Spain for the assessment of anxiety and fear of COVID-19 in this population.

## Data availability statement

The raw data supporting the conclusions of this article will be made available by the authors, without undue reservation.

## Ethics statement

This study received the approval of the Biomedical Research Ethics Committee of the province of Huelva in the resolution approved at the session held on January 19, 2021 and recorded in Minutes 01/21 (PI036/20). Adherence to this resolution was also obtained from the Biomedical Research Ethics Committee of the province of Malaga on November 26, 2021. The studies were conducted in accordance with the local legislation and institutional requirements. The participants provided their written informed consent to participate in this study.

## Author contributions

FM-V, LR-D, JG-S, FF-C, RA-C, JV-L, and JF-R: conceptualization, formal analysis, investigation, methodology, resources, software, validation, visualisation, writing—original draft, and writing—review and editing. FM-V, LR-D, and JV-L: data curation, project administration, and supervision. All authors contributed to the article and approved the submitted version.

## Conflict of interest

The authors declare that the research was conducted in the absence of any commercial or financial relationships that could be construed as a potential conflict of interest.

## Publisher’s note

All claims expressed in this article are solely those of the authors and do not necessarily represent those of their affiliated organizations, or those of the publisher, the editors and the reviewers. Any product that may be evaluated in this article, or claim that may be made by its manufacturer, is not guaranteed or endorsed by the publisher.
