# Supplementary material for: Adaptation and psychometric study of the scale for the measurement of fear and anxiety of COVID-19 disease in pregnant women (AMICO_Pregnant)
Source: Front Public Health. 2023 Sep 21;11:1225822. doi: 10.3389/fpubh.2023.1225822 (PMC10552539; doi:10.3389/fpubh.2023.1225822)
Supplement: Supplementary file 1 [file Data_Sheet_1.docx]

**Supplementary File 1. AMICO_Pregnant Scale.**

**Item 1.** I am very afraid of COVID-19.

**Item 2.** Thinking about COVID-19 causes me distress.

**Item 3.** I am very worried about getting COVID-19 for my health and my baby's health.

**Item 4.** The COVID-19 disease can be life threatening, and this worries me for my health or my baby's health.

**Item 5.** I get very nervous when I think about COVID-19.

**Item 6.** I feel nervous or anxious when watching news or stories about COVID-19 in social networks and other media.

**Item 7.** I have trouble sleeping if I think I might get COVID-19.

**Item 8.** My pulse races if I have been in close contact with someone at risk of being infected.

**Item 9.** Contradictory information about COVID-19 in the media and social networks makes me feel anxious.

**Item 10**. I am assaulted by negative thoughts when I hear or read news related to COVID-19.

**Item 11.** I am worried that a family member or friend might contract COVID-19.

**Item 12.** I am worried about how long the pandemic will last.

**Item 13.** When someone coughs near me, or I consider he or she is too close to me, I am afraid of getting infected.

**Item 14.** I am worried about being close to or assisting a person that has or may have COVID-19.

**Item 15.** I feel sad or weak when I think about the disease and the possibility of infecting myself or my baby.

**Item 16.** I feel anxious about leaving home, or thinking about it, to fulfil my daily duties (work, family, etc.) due to the current pandemic situation.

-------

The range of scores in each question is from 1 to 10, where 1 is the lowest level and 10 the highest possible level.
